# Suppression of literal meaning in single and extended metaphors

**DOI:** 10.3389/fpsyg.2023.1135129

**Published:** 2023-03-15

**Authors:** Camilo R. Ronderos, Ingrid Lossius Falkum

**Affiliations:** Department of Philosophy, Classics, History of Art and Ideas, University of Oslo, Oslo, Norway

**Keywords:** relevance theory, extended metaphors, metaphor comprehension, experimental pragmatics, figurative language

## Abstract

Within Relevance Theory, it has been suggested that extended metaphors might be processed differently relative to single metaphoric uses. While single metaphors are hypothesized to be understood *via* the creation of an *ad hoc* concept, extended metaphors have been claimed to require a switch to a secondary processing mode, which gives greater prominence to the literal meaning. Initial experimental evidence has supported a distinction by showing differences in reading times between single and extended metaphors. However, beyond potential differences in comprehension speed, Robyn Carston’s ‘lingering of the literal’ account seems to predict qualitative differences in the interpretative mechanisms involved. In the present work, we test the hypothesis that during processing of extended metaphors, the mechanisms of enhancement and suppression of activation levels of literal-related features operate differently relative to single metaphors. We base our work on a study by Paula Rubio-Fernández, which showed that processing single metaphors involves suppressing features related exclusively to the literal meaning of the metaphoric vehicle after 1000 milliseconds of encountering the metaphor. Our goal was to investigate whether suppression is also involved in the comprehension of extended metaphors, or whether the ‘lingering of the literal’ leads to continued activation of literal-related features, as we take Carston’s account to predict. We replicate existing results, in as much as we find that activation levels of literal-related features are reduced after 1000 milliseconds. Critically, we also show that the pattern of suppression does not hold for extended metaphors, for which literal-related features remain activated after 1000 milliseconds. We see our results as providing support for Carston’s view that extended metaphor processing involves a prominent role of literal meaning, contributing towards explicating the links between theoretical predictions within Relevance Theory and online sentence processing.

## Introduction

The cognitive mechanisms responsible for metaphor comprehension have been the focus of much research throughout the last several decades (for reviews see Holyoak and Stamenković, 2018; Pouscoulous and Dulcinati, 2019). One reason for the sustained interest in this line of work is the apparent change in meaning that words undergo when used metaphorically. Take example (1):

*1. John doesn’t like physical contact, and even his girlfriend finds it difficult to come close to him. She feels rejected by his distant attitude every time he sees her. John is a cactus.*

It is clear that the word *cactus* is not used to refer to a type of plant, but to John’s distant demeanor. But how does a comprehender go from understanding *cactus* as a plant to understanding it as a personality trait? Two related theories provide an answer this question by viewing metaphor comprehension as a form of category extension: Glucksberg’s Dual Reference Account (Glucksberg, 2001, 2008), and Sperber and Wilson’s Deflationary Account (Sperber and Wilson, 2008), which is embedded in the larger framework of Relevance Theory (Wilson and Sperber, 2012). Broadly speaking, both accounts state that comprehenders understand nominal metaphors as (1) by inferentially adjusting the meaning of the metaphoric vehicle (*cactus*) on the basis of the salient interpretative dimensions provided by the metaphoric topic (*John*), and crucially, encyclopedic information associated with the metaphor vehicle (*cactus*) together with the relevant context. Once this occurs, the topic is understood as being a member of an occasion-specific (*ad hoc*) category represented by the vehicle (McGlone and Manfredi, 2001; Glucksberg, 2003; Rubio Fernández, 2007; Sperber and Wilson, 2008). This idea borrows from previous work on the comprehension of *ad hoc* categories, which suggests that people are in general quite good at picking out the potential members of a newly created group (e.g., *things to bring to a picnic*, Barsalou, 1983). In this way, understanding so-called nominal metaphors [‘A is a B’ constructions such as (1)] is similar to understanding category inclusion statements such as *Papaya is a fruit*.

The relevance-theoretic view extends beyond the analysis of simple nominal metaphors. The view is that metaphors in general are understood on the basis of the same interpretative mechanism as other forms of lexical interpretation (hence the *deflationary* character of the account). According to the theory, lexical interpretation typically involves modulation of encoded word meanings, where *ad hoc* concepts are constructed in accordance with the hearer’s occasion-specific expectations of relevance, based on the encoded concepts, a set of associated encyclopedic assumptions, and information derived from the utterance context (Wilson and Carston, 2007). *Ad hoc* concepts can either be more specific (‘narrower’) or more general (‘broader’) than the word’s encoded meaning (as it is assumed to be stored in the mental lexicon). Critically, metaphors are said to result in both narrowing and broadening of the encoded meaning (Wilson and Carston, 2006; Carston, 2010). For example, in (1), *cactus* is broader than the encoded meaning because it includes a type of ‘prickly’ people, which the encoded concept excludes. It is also narrower than the encoded meaning because it excludes cacti without spikes (e.g., the spike-less peyote plant).

Broadening and narrowing can be thought of in terms of property promotion and demotion (Carston, 2002). According to Carston (2002), mentally stored concepts provide a memory link to three types of information: logical content, encyclopedic content, and lexical properties. Logical content is meaning-constituent (e.g., Cactus is a kind of plant), whereas encyclopedic content represents general world knowledge we associate with a specific concept (e.g., Cacti typically have spikes, they grow in the desert, etc.). During lexical modulation, some properties associated with the encoded concept are promoted whereas others are demoted. Property promotion and demotion can be conceptualized in psychological terms as the degree of activation of a particular property: A promoted property is highly activated, whereas a demoted property is not (e.g., Rubio Fernández, 2007). This would mean that, when constructing the (metaphorical) *ad hoc* concept CACTUS*,^1^ certain encyclopedic features that are associated with the encoded meaning of *cactus* become highly activated (e.g., the fact that cacti have spikes), whereas those that are not relevant for the construction of the *ad hoc* concept have a substantially lower degree of activation (e.g., that cacti are a kind of plant).

Narrowing and broadening in the form of construction of *ad hoc* concepts are the outcomes of the interpretative process (Wilson and Carston, 2006; Carston, 2010). However, thinking of them as the degree of activation of encyclopedic features provides a link to the cognitive mechanisms potentially involved in metaphor comprehension, such as the mechanisms responsible for the suppression and enhancement of activation levels. Gernsbacher and Faust (1991) state that language comprehension in general is enabled by the enhancement and suppression of the activation levels of memory nodes. In this view, enhancement regulates the increase of activation of relevant information and suppression regulates the reduction of activation of irrelevant information. This led Gernsbacher et al. (2001) and, subsequently, Rubio Fernández (2007), to derive explicit hypotheses for category extension theories in terms of suppression and enhancement of associated features during metaphor comprehension. When processing a metaphor such as (1)–once the interpretative dimensions are made salient by the context and the metaphoric topic (that John is human and that his personality is being discussed)–comprehenders adjust the lexically encoded meaning of *cactus*. They do this by suppressing the activation of features that mismatch these dimensions (and are thus irrelevant for the unfolding interpretation, e.g., that cacti are plants), and by enhancing the activation of those that match (and are thus relevant for the interpretation, e.g., that cacti are prickly).

To test these claims, Gernsbacher et al. (2001) showed participants prime sentences that were either literal or metaphoric [*That large hammerhead is a shark* (literal), *that defense lawyer is a shark* (metaphoric)]. Participants then read and verified sentences that included words representing properties that were relevant or irrelevant for the *ad hoc* category [*sharks are tenacious* (relevant), *sharks are good swimmers* (irrelevant)]. The results showed that participants were faster to verify sentences about a metaphor-relevant feature following the metaphoric prime compared to when the sentence followed a literal prime. Conversely, verifying sentences describing a metaphor-irrelevant property was less costly when these followed a literal prime than when they followed a metaphoric prime.

Rubio Fernández (2007) provided further evidence using a cross-modal priming paradigm. In it, participants first heard a novel metaphor (*John is a cactus*) and, immediately after hearing the vehicle, read a target word and performed a lexical decision task. Critically, target words were shown at three possible intervals (0, 400 and 1,000 milliseconds after the end of the metaphor). Target words were either ‘literal’ superordinates of the metaphoric vehicle (and therefore irrelevant for the construction of the *ad hoc* category, e.g., *plant*) or distinctive properties at the core of the metaphoric meaning (relevant for constructing the *ad hoc* category, e.g., *spike*). The results showed that in the two earliest intervals (0 ms and 400 milliseconds), superordinates and distinctive properties were similarly activated. However, in the last interval (1,000 milliseconds), only distinctive properties remained active, while superordinates appeared to be suppressed. The results of both of these studies suggest that, during metaphor comprehension, metaphor-related features (i.e., the distinctive features) are suppressed while literal-related ones (i.e., the superordinates) remain active [for at least 1,000 milliseconds after the metaphor has been understood, according to Rubio Fernández (2007)]. This supports the *ad hoc* concept account by showing how comprehending a metaphor brings about the modulation of the encoded meaning of the metaphoric vehicle.

Despite the above-mentioned evidence, the issues of the mechanisms involved during comprehension and the validity of the *ad hoc* concept account are far from settled. This is in part due to the existence of rivaling theories that also have some empirical support (e.g., the structure mapping view of Gentner and Bowdle, 2008). It is also due to existing experimental evidence being rather limited in scope. The experiments discussed thus far focused on so-called single nominal metaphors (of the ‘X is a Y’ type). However, metaphors can come in all shapes and sizes. They can be expressed through verbs (*The sunflower danced in the sun*) or adjectives (*Miguel has a colorful personality*), for example. Examining a wide variety of cases, as argued by Holyoak and Stamenković (2018), is essential for assessing the robustness of a theory whose goal should be to account for the mechanisms behind metaphor processing independently of the morphosyntactic properties of the metaphoric vehicle. Steps have been taken in this direction, with various studies investigating the processing of non-nominal metaphors in recent years (e.g., Cardillo et al., 2012; Ronderos et al., 2021, 2022).

Besides the focus on nominal metaphors, an overwhelming majority of studies have looked at ‘single’ metaphors only, i.e., metaphors with a unique metaphoric vehicle. As a contrast to this, consider the case in which example (1) is slightly modified into (2):

*2. John doesn’t like physical contact, and even his girlfriend finds it difficult to come close to him. She feels pricked by his thorny attitude every time he sees her. John is a cactus*

In (2), the words *thorny* and *pricked* denote properties that are associated with the concept encoded by the word *cactus*, and thus their literal meanings are semantically related. Importantly, these words are used metaphorically in a way consistent with the metaphor in the sentence *John is a cactus*. As a whole, this passage constitutes what is known as an extended metaphor (Carston, 2010; Rubio-Fernández et al., 2016).

Carston (2010) suggests that extended metaphors might pose a problem for the relevance-theoretic analysis developed examining single metaphors only. This is because the mechanism proposed by Sperber and Wilson’s deflationary account of metaphor in terms of *ad hoc* concept construction is a form of local meaning adjustment: each time a metaphoric vehicle is encountered (e.g., *cactus*), an *ad hoc* concept is created that differs from the encoded meaning in that it has been broadened (and typically also narrowed) (e.g., creating the *ad hoc* category CACTUS*). For an extended metaphor such as (2), this means that, upon encountering the words *thorny*, *pricked* and *cactus*, comprehenders would have to suppress literal features irrelevant to the metaphoric meaning each time. This would occur despite the fact that these three words are clearly related to each other and their literal meaning is likely to be highly activated given backwards and forward priming. The local lexical adjustment mechanism would result, according to Carston (2010), in a demanding and effortful process (but see Wilson, 2018, for a different perspective on this issue). Instead, Carston (2010) suggests, comprehenders might begin to maintain–through metarepresentation–the literal meaning of extended metaphors as a whole (because of how the literal meaning of the different vehicles ‘lingers’ and is therefore highly activated) and subject this literal interpretation to slower, broader inferences after the entire expression has been understood. In terms of online metaphor processing, the account proposed by Carston (2010) could be said to make one general prediction: metaphoric vehicles comprehended as part of extended metaphoric passages should be processed differently than the same vehicles encountered as stand-alone metaphors. There is some evidence to this effect that pre-dates Carston’s ‘lingering of the literal’ account. Keysar et al. (2000) had participants read vignettes that included multiple metaphoric vehicles stemming from the same conceptual domain. They found that when the metaphors were novel (Experiment 2), target metaphoric sentences were read faster when preceded by related metaphoric vehicles relative to when the previous sentences contained no metaphors whatsoever. A similar result using the same paradigm was also reported by Thibodeau and Durgin (2008).

In a more explicit test of the ‘lingering’ account, Rubio-Fernández et al. (2016) used self-paced reading (Experiment 1), eye-tracking during reading (Experiment 2) and cued recall (Experiment 3) to examine processing differences between single and extended metaphors as well as literal controls. They found that participants took longer to read single metaphors relative to extended metaphors and literal controls (Experiment 1). In Experiment 2, they found that extended metaphors and literal controls were read similarly fast in an early reading measure (i.e., first-pass reading), whereas single metaphors were found to take longer to read. However, the difference between single and extended metaphors seemed to dissipate in later reading measures (i.e., total reading times). The authors interpret this as supporting Carston’s view of a distinction between the two types of metaphor: Extended metaphors are first read as fast as a literal utterance (thus suggesting an early advantage in comprehension time for extended relative to single metaphors), and the late derivation of inferences in the extended metaphors case results in more effortful processing of extended vs. single metaphors in the latest moments of processing.

Despite the fact that this pattern of findings suggests a difference between both types of metaphors, it is unclear whether this difference is a *qualitative* or a *quantitative* one. In other words, it could be that extended metaphors are subjected to the same mechanisms as single metaphors but simply undergo the process of lexical modulation faster because of low-level priming brought on by the previously understood metaphors. This is akin to the view put forth by Wilson (2018), p. 195, who claims that differences between single and extended metaphors has more to do with a ‘lingering of linguistic form’ than a ‘lingering of literal meaning’, where the accumulation of metaphorical vehicles with related encoded meanings “will encourage some [hearers] to pay more attention to the exact wording of the [utterance] and search for further implications activated by the encoded meaning.” Though Wilson (2018) does not explicate her view of the ‘lingering of the linguistic form’ in processing terms, one could explain the faster processing observed for extended compared to single metaphors as resulting not from a qualitative difference in processing between the two types of metaphor but from low-level semantic priming stemming from processing various related metaphoric vehicles in a row [e.g., *pricked* and *thorny* in (1)]. This priming facilitates access to the entry in the mental lexicon of the subsequent related vehicle (*cactus*). Once the lexical entry has been accessed, processing continues normally, with comprehenders creating a new *ad hoc* category (CACTUS*). This would amount to a difference in *degree* of activation relative to single metaphors, and not a difference in kind. Extended metaphors would be comprehended faster than single metaphors but making use of the same mechanisms.

Alternatively, it could be that processing differences between extended and single metaphors are truly due to qualitative differences in the underlying mechanisms, as suggested by Carston (2010). In Carston’s view, the persistent high activation of the closely related literal meanings of the metaphoric vehicles makes the creation of *ad hoc* categories for every single one of them too effortful. Instead, comprehenders metarepresent the literal meaning of the expressions throughout the processing of the extended metaphor. In processing terms, this would not only lead to differences in comprehension speed, but should also result in the involvement of different comprehension mechanisms: Single metaphors are processed *via* the construction of *ad hoc* categories (following the standard relevance-theoretic account), while extended metaphors are processed literally, with a literal representation of the entire passage being maintained and metarepresented even as metaphoric inferences are drawn. However, it is not entirely clear whether Carston’s account would actually predict faster processing of extended metaphors, as suggested by Rubio-Fernández et al. (2016), or in *slower* processing due to metarepresentation of the literal meaning and derivation of a range of weak implicatures.

The goal of the current work is to test these two alternatives by examining the role of enhancement and suppression of the activation levels of literal features during processing of single and extended metaphors. As previously mentioned, others have suggested that when understanding a metaphor, features related exclusively to the literal meaning of a vehicle (what we refer to as ‘literal-related features’) are suppressed, while those related to the resulting metaphoric meaning (which we dub ‘metaphor-related features’) enhanced (Gernsbacher et al., 2001; Rubio Fernández, 2007). How should enhancement and suppression play out during processing of extended metaphors? One possibility is that the differences in comprehension effort for single relative to extended metaphors reported by Keysar et al. (2000), Thibodeau and Durgin (2008), and Rubio-Fernández et al. (2016) result in baseline differences in activation levels for both literal-related and metaphor-related features: being exposed to related metaphors facilitates lexical access to the subsequent related metaphoric vehicle, and therefore the recognition of both types of features is made easier at all time intervals. This would suggest that suppression and enhancement operate in basically the same way for extended metaphors as they do for single metaphors. They simply operate faster, in line with the view that one difference between the two types of metaphors is that extended metaphors, but not single ones, involve low-level priming of linguistic form. Another possibility would be that that there are differences in how suppression and enhancement unfold over time: While literal-related features are suppressed after around 1,000 milliseconds and metaphor-related features remain active in the case of single metaphors (Rubio Fernández, 2007), it could be that the mechanism of suppression is suspended when processing extended metaphors. This would result in sustained activation for literal-related features at different time intervals after processing the metaphor. This process would be in line with the view that literal meaning is metarepresented during the comprehension of extended metaphors (Carston, 2010).

To be clear, both alternatives are in principle compatible with a processing speed advantage for extended relative to single metaphors: The ‘lingering of linguistic form’ can be interpreted as a low-level priming effect that facilitates the retrieval of subsequent related metaphoric vehicles, whereas the ‘lingering of the literal’ leads the expression as a whole to be initially processed literally, without engaging in the construction of *ad hoc* concepts. However, it seems that only Carston’s view would predict qualitative differences between single and extended metaphors in how suppression and enhancement of activation levels of literal features unfold over time. If the literal meanings of the metaphorical vehicles are metarepresented throughout the processing of the extended metaphor, it is likely that also features related to these literal meanings (and which are irrelevant to the metaphorical meanings) retain a high activation level, or at least are not suppressed to the same extent as if *ad hoc* concepts were created for each of the metaphorical vehicles. To test this key difference, we adapted Rubio Fernández (2007) seminal paradigm to a web-based experiment, and present the results of our study in the following section.

## Method

### Participants

We recruited a total of 460 participants *via* the online recruitment platform Prolific. Participants were all monolingual native speakers of American English between the ages of 18 and 35. They all had an IP-address from the United States during time of testing and reported being right-handed. Of these, 3 were excluded because of a technical problem. Of the remaining 457, 47 were excluded for not meeting the minimum accuracy requirement (i.e., achieving at least 70% accuracy in the lexical decision task across critical and filler trials). This left the total number of participants at 410.

### Materials and design

The starting point of our investigation was the experiment conducted by Rubio Fernández (2007). Since we intended to adapt the original experiment to a web-based task, we made three main adjustments. First, instead of using a cross-modal paradigm (where the prime is heard by participants and the target sentence read on the screen), we presented both primes and targets visually. This was done given the reduced amount of experimental control in a web-based experiment. For example, it was not possible for us to know if participants would use headphones or speakers or if they would be listening to music while completing the task. Therefore, presenting both prime and target in the written form seemed like an appropriate way to reduce noise and make sure that they were both understood. Second, we chose to use only a subset of the items used by Rubio Fernández (2007) to keep the experiment as short as possible and thus maximize the quality of the data collected from the online participants, following recommendations by Futrell (2012). We used 8 of the critical items from Rubio Fernández (2007) as our target items, and another group of 8 as fillers. Third, we reduced the number of Inter-Stimulus Intervals (ISI) tested relative to the original experiment from three (0, 400 and 1,000 milliseconds) to two (0 and 1,000 milliseconds). This was done to keep the number of experimental conditions to a minimum.

**Figure 1:**
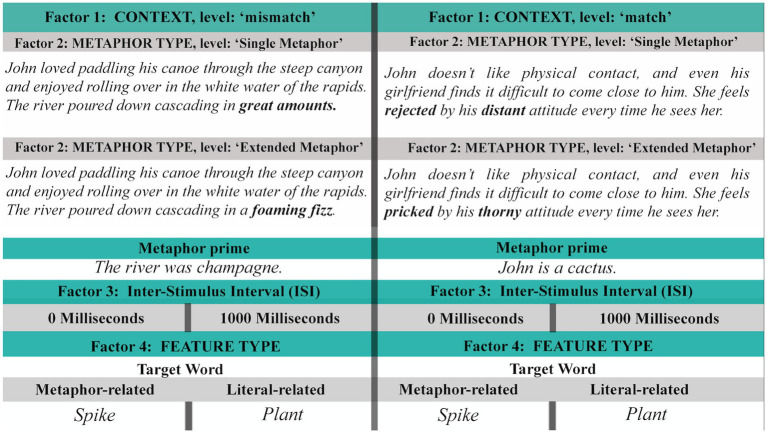
Example of a critical trial in all conditions.

After making these adjustments we adapted the materials in order to create an extended-metaphoric version of each item. To do this, we added an additional context sentence prior to the nominal metaphor. For extended metaphors, this context sentence included additional metaphoric vehicles that drew from the same conceptual domain as the nominal metaphor. For single metaphors, the additional context sentence was a literal equivalent. This is exemplified in sentences (1) and (2), with all conditions reproduced in Figure 1 below.

The experiment was programmed using the PCIbex experimental software (Zehr and Schwarz, 2018) and had a 2X2X2X2 design (as seen in Figure 1): Participants first read a context (in a single or extended metaphor set up) and a metaphor prime that was either related or unrelated to the target word they would see afterwards (factor 1: CONTEXT, levels: ‘match’ vs. ‘mismatch’). This factor ensures that we have a baseline measure of the lexical decision time for each word in the ‘match’ conditions: Every target word appeared following a single and extended matching or mismatching metaphor. In other words, responses to target words appeared in the absence of any potential semantic priming (i.e., when the metaphor prime is completely unrelated to the target, in the ‘mismatch’ level), as well as following a corresponding related single or extended metaphor (i.e., when the metaphor prime is critically related to the target, in the ‘match’ level). Each critical item was paired with another one to create the ‘mismatch’ conditions, so that every target word and every metaphor appeared equally in ‘match’ and ‘mismatch’ conditions across lists. The metaphor primes read were either instances of single or extended metaphors, depending on critical words being altered accordingly in the context (factor 2: METAPHOR TYPE, levels: ‘single metaphor’ vs. ‘extended metaphor’, see words in bold in Figure 1). After reading the metaphors, participants were forced to wait either 1,000 ms. Or to directly continue to the lexical decision task (factor 3: ISI, levels: 0 and 1,000 milliseconds). Finally, participants read the target words and performed a lexical decision task. Target words were either related to the irrelevant encoded literal meaning of a metaphoric vehicle only, or to the vehicle’s relevant metaphoric meaning (factor 4: FEATURE TYPE, levels: ‘literal-related vs. ‘metaphor-related, or they were irrelevant to both, as in the ‘mismatch’ condition). As mentioned, we used an additional 8 of the original critical items of Rubio Fernández (2007) as fillers. These consisted of metaphoric primes and plausible English pseudo-words as targets. The pseudo-words were created using the online pseudo-word generator Wuggy^2^, designed for use in psycholinguistic experiments. Both filler and critical trials in the experiment were metaphors, in line with the set-up used by Rubio Fernández (2007). Half of the filler items were extended metaphors and half were single metaphors. Half of the target pseudo-words were presented with an ISI of 1,000 milliseconds, with the other half having an ISI of 0 milliseconds. We created 16 experimental lists using a latin-squared design, distributing conditions in a balanced way across lists. However, since we only had 8 critical items, it was not possible for one participant to see all combinations of conditions in a single experimental list, given that this would have required at least 16 items. Instead, each participant saw 8 critical items in 8 different conditions, ensuring that they saw each level of each factor at least 4 times (across items), with all conditions evenly distributed through the 16 lists. This made our design a between-subjects one regarding the four-way interaction only, and a within-subjects design regarding all other comparisons. The master list including all 16 list combinations, together with all critical and filler items as well as the data and analysis script can be found on the project’s OSF page: https://osf.io/eayj7/.

### Procedure

Participants first read the instructions of the experiment. They were then asked to make sure they were in a quiet environment, and were instructed to position their index fingers above the F and J key, and leave their thumbs over the SPACEBAR. In each trial, participants first read the context sentences. They had to press the SPACEBAR to continue once they finished reading what was presented to them. At this point, they were presented with the metaphor prime, which was always a nominal metaphor of the form ‘X is a Y’. The metaphor prime was presented on screen until participants pressed the SPACEBAR, which they were told to press as soon as they had finished reading. Participants had to wait at least 1,500 milliseconds before being allowed to continue, and were required to press the SPACEBAR before 4,000 milliseconds after the prime metaphor was presented. If it took them more than 4,000 milliseconds to read the metaphor prime, the trial automatically exited and a text appeared on screen prompting them to be faster, without any data for this particular trial being recorded. This was done to discourage participants from reading the metaphors and waiting too long before moving onto the lexical decision task. Once participants pressed the SPACEBAR within the time limits, they were asked to decide whether a word presented onscreen was a real word of English or not. They had to use the F (‘not a real word’) and J (‘real word’) keys to make their decision. They had a maximum of 2000 milliseconds to press a key. If they failed to respond within this time, the trial would automatically end, participants would be asked to respond faster next time and the next trial would begin. Participants first went through two practice trials before the actual experiment began. They then saw filler and target items, which appeared in randomized order.

### Analysis

To analyze the data we used the R programming language (R Core Team, 2020) and R-Studio (RStudio Team, 2020). For data processing, visualization and analysis, we used the following packages: ggplot2 (Wickham, 2016), lme4 (Bates et al., 2007), Rmisc (Hope, 2013), MASS (Ripley et al., 2013), dplyr (Wickham et al., 2020), DoBy (Højsgaard, 2012), papaja (Aust and Barth, 2017), here (Müller, 2017), and afex (Singmann et al., 2020).

Prior to inferential analysis, we removed all participants who failed to accurately respond to the lexical decision task at least 70% of the time (across critical and filler trials). We also removed all critical trials for which participants gave a wrong answer. We then log-transformed the reaction times of the lexical decision task given that the residuals of a model using raw-reaction times were not normally distributed, and used log-milliseconds as our dependent variable.

We fitted a linear, mixed-effects model to the log-transformed data following the recommendations of Barr et al. (2013). The model included the four factors (METAPHOR TYPE, CONTEXT, FEATURE TYPE and ISI) and all possible interactions as fixed effects. Three factors (CONTEXT, FEATURE TYPE and ISI) had an ANOVA-style, sum-contrast coding, whereas the fourth factor METAPHOR TYPE was treatment-contrast coded, with the factor ‘single metaphors’ as the baseline. This allowed us to assess the three-way interaction between CONTEXT, FEATURE TYPE and ISI in the single metaphor case. This model was then re-fitted using the level ‘extended metaphors’ of the METAPHOR TYPE factor as the baseline. This second version of the model allowed us to examine the three-way interaction between CONTEXT, FEATURE TYPE and ISI for single metaphors, as well as the four-way interaction between METAPHOR TYPE, CONTEXT, FEATURE TYPE and ISI.

The random effects structure included random intercepts by items and by subjects. It also included random slopes for all factors and all possible interactions by items. The by-subjects random effects included slopes for all factors and all two- and three-way interactions.

### Predictions

Our predictions relate both to the comparison between single and extended metaphors, as well as to the replication of the original results of Rubio Fernández (2007). Rubio Fernández (2007) reported a loss of activation for superordinates (what we refer to as literal-related features) between 400 and 1,000 milliseconds relative to the activation levels of distinctive properties (referred to as metaphor-related features in the current study), which remained activated even after 1,000 milliseconds. Since we did not include a 400 millisecond level, we took the observed difference between 0 and 1,000 milliseconds in Rubio Fernández’s experiment as the basis for the replication. For this reason, we considered that if the three-way interaction between CONTEXT, FEATURE TYPE and ISI was significant for the case of single metaphors, it would suggest that the activation levels of literal-related and metaphor-related features change as a function of time after processing single metaphors, in line with the original findings of Rubio Fernández (2007). If, on the other hand, this interaction is not significant, it would be at odds with the results of the original study. Our second prediction refers to the comparison between single and extended metaphors. Recall that we take the ‘lingering of the literal’ account proposed by Carston (2010) to predict qualitative differences in terms of the mechanisms involved in metaphor processing: Single metaphors are understood *via* the lexical modulation of the metaphoric vehicle, while during the comprehension of extended metaphors the literal meaning of the metaphor is maintained as a whole, with inferences projected later downstream in the form of an array of weak implicatures. In terms of the activation levels of literal-related features, the ‘lingering of the literal’ could translate to enhanced activation of these associated features given how both the literal meanings of the multiple related vehicles and features that are associated with them prime each other. This would mean that the three-way interaction between CONTEXT, FEATURE TYPE and ISI should not be significant for extended metaphors: the way in which activation levels of metaphor-related and literal-related features changes over time (relative to the unrelated baseline provided by the ‘mismatch’ conditions) should not be different from one another. This pattern should be accompanied by a significant four-way interaction between all four factors. This would suggest that while literal-related features are suppressed with time when understanding single metaphors (supporting the lexical modulation of the meaning of the vehicle), these associated features would remain activated in the extended metaphor case, where the literal meaning would be metarepresented as a whole.

Alternatively, if we fail to find a significant four-way interaction and instead find similar three-way interactions between CONTEXT, FEATURE TYPE and ISI for both single and extended metaphors, it would suggest that the underlying mechanisms involved when processing single and extended metaphors are similar, contra Carston (2010).

### Results

**Figure 2:**
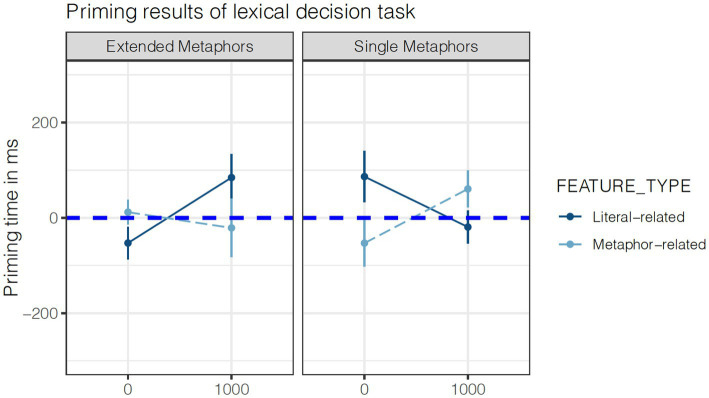
Activation pattern of literal-related and metaphor-related features. ‘Priming time’ was calculated by subtracting the ‘match’ level of the factor CONTEXT from the ‘mismatch’ level. This yielded the difference in milliseconds between processing the target word in the presence vs. absence of a related metaphor. Error bars show Standard Errors.

The pattern of results is shown in Figure 2, while the summary of the model’s output is shown in Tables 1, 2. Figure 2 shows the results in terms of ‘priming time’ for illustration purposes only, following the original reporting of results in Rubio Fernández (2007). This measure was calculated by subtracting the average values of response times in each ‘match’ from its corresponding ‘mismatch’ condition of the factor CONTEXT by items. By doing this, we obtained an estimate of the ‘priming time’ of each target word relative to the control baseline: Positive numbers represent a facilitatory effect (i.e., a positive priming effect on the lexical decision task of the target word), whereas negative numbers represent an inhibitory effect (i.e., a negative priming effect).

**Table 1:** Summary of regression model output with ‘single metaphors’ as baseline condition.

Term	*β*	95% CI	*t*	df	*p*
ISI	0.06	[0.01, 0.11]	2.42	12.82	0.031
METAPHOR TYPE	−0.01	[−0.05, 0.02]	−0.79	16.18	0.442
FEATURE TYPE	0.02	[−0.05, 0.09]	0.63	47.50	0.533
CONTEXT	0.03	[−0.01, 0.07]	1.63	17.02	0.122
ISI*METAPHOR TYPE	−0.04	[−0.10, 0.02]	−1.41	627.06	0.160
ISI*FEATURE TYPE	0.01	[−0.07, 0.09]	0.29	347.83	0.769
METAPHOR TYPE*FEATURE TYPE	−0.06	[−0.17, 0.05]	−1.07	369.94	0.286
ISI*CONTEXT	0.01	[−0.07, 0.09]	0.27	344.06	0.785
METAPHOR TYPE*CONTEXT	−0.05	[−0.11, 0.00]	−1.87	339.68	0.062
FEATURE TYPE*CONTEXT	0.01	[−0.07, 0.09]	0.28	15.98	0.787
ISI*METAPHOR TYPE*FEATURE TYPE	−0.05	[−0.17, 0.07]	−0.81	645.37	0.421
ISI*METAPHOR TYPE*CONTEXT	−0.09	[−0.22, 0.03]	−1.47	17.69	0.159
ISI*FEATURE TYPE*CONTEXT	0.26	[0.11, 0.42]	3.29	343.06	0.001
METAPHOR TYPE*FEATURE TYPE*CONTEXT	0.04	[−0.06, 0.15]	0.80	342.91	0.425

METAPHOR TYPE was treatment-contrast coded, all other factors were sum-contrast coded.

**Table 2:** Summary of regression model output with ‘extended metaphors’ as baseline condition.

Term	*β*	95% CI	*t*	df	*p*
ISI	0.02	[−0.04, 0.08]	0.69	16.61	0.499
METAPHOR TYPE	0.01	[−0.02, 0.05]	0.75	22.62	0.461
FEATURE TYPE	−0.03	[−0.10, 0.03]	−1.01	541.60	0.312
CONTEXT	−0.02	[−0.06, 0.02]	−0.95	19.47	0.352
ISI*METAPHOR TYPE	0.04	[−0.02, 0.10]	1.43	630.65	0.154
ISI*FEATURE TYPE	−0.04	[−0.12, 0.05]	−0.79	290.65	0.433
METAPHOR TYPE*FEATURE TYPE	0.05	[−0.07, 0.17]	0.76	64.33	0.449
ISI*CONTEXT	−0.08	[−0.16, 0.00]	−1.95	386.61	0.052
METAPHOR TYPE*CONTEXT	0.05	[0.00, 0.11]	1.82	368.06	0.070
FEATURE TYPE*CONTEXT	0.05	[−0.03, 0.14]	1.23	19.00	0.234
ISI*METAPHOR TYPE*FEATURE TYPE	0.05	[−0.07, 0.16]	0.76	646.20	0.449
ISI*METAPHOR TYPE*CONTEXT	0.09	[−0.02, 0.20]	1.55	376.52	0.121
ISI*FEATURE TYPE*CONTEXT	−0.09	[−0.25, 0.07]	−1.11	385.36	0.266
METAPHOR TYPE*FEATURE TYPE*CONTEXT	−0.04	[−0.15, 0.07]	−0.71	371.25	0.477
ISI*METAPHOR TYPE*FEATURE TYPE*CONTEXT	0.35	[0.12, 0.57]	3.04	379.25	0.003

METAPHOR TYPE was treatment-contrast coded, all other factors were sum-contrast coded

Table 1 shows the results for single metaphors (i.e., with ‘single metaphors’ as the baseline level for the factor METAPHOR TYPE). Here, we find a three-way interaction between CONTEXT, FEATURE TYPE and ISI (*t-*value = 3.3, *p* < 0.005). As can be seen in Figure 2, this interaction suggests that in the one-second difference in ISI, literal-related features are significantly reduced in activation relative to metaphor-related features (when comparing lexical decision times following the metaphor primes to lexical decision times of the same target words following unrelated controls). The results summarized in Table 2, which show the overall pattern for extended metaphors (i.e., with ‘extended metaphors’ as the baseline level for the factor METAPHOR TYPE), paint a different picture. Here, we failed to find a significant three-way interaction between CONTEXT, FEATURE TYPE and ISI (*t*-value = 1.1, *p* = 0.26). Crucially, there was a significant four-way interaction between all factors (*t*-value = 3, *p* < 0.005), suggesting that the pattern of activation of literal-related and metaphor-related features is different for extended relative to single metaphors. As Figure 2 suggests, it does not seem to be the case that literal-related features are suppressed with the change in ISI, in opposition to what seems to happen during processing of single metaphors.

## General discussion

In the current work, we set out to test the potential implications of Robyn Carston’s ‘lingering of the literal’ account for the processing of extended metaphors. The account postulates a difference in processing strategies between extended and single metaphors. Processing single metaphors, according to the standard view within Relevance Theory, depends on the rapid construction of *ad hoc* categories. However, according to Carston (2010), relying on this mechanism might turn out to be overly strenuous for comprehenders when faced with an extended metaphor. This is because an extended metaphoric passage has multiple metaphoric vehicles that share the same literal conceptual domain. These multiple vehicles likely reinforce each other’s literal meaning, leading comprehenders to maintain a representation of the literal meaning of the expression as a whole instead of relying on the lexical modulation of each vehicle individually. This account can explain why it has been consistently reported that there is a difference in processing between understanding metaphors preceded by other metaphors from the same conceptual domain relative to understanding the same metaphors presented in isolation (see also Gentner et al., 2001, for an alternative account). These empirical findings, however, can be explained *via* other accounts as well. Within Relevance Theory, for example, Wilson (2018) has claimed that exposure to extended metaphors brings about a ‘lingering of linguistic form’. One possibility is that this involves a low-level facilitation effect that does not require the literal meaning of an expression to be meta-represented and does not bring about a qualitative difference in processing extended relative to single metaphors. In other words, it could be that comprehenders rely on the same mechanisms for understanding single and extended metaphors, and simple low-level priming that facilitates lexical access explains differences in comprehension time without requiring different mechanisms. Therefore, because existing empirical findings are compatible with multiple accounts, it is necessary to produce a stronger test of Carston’s account. Specifically, one that can help determine whether the difference in processing single and extended metaphors is really caused by qualitative differences in the underlying comprehension mechanisms. The present work is a step in this direction. Based on the study by Rubio Fernández (2007), we set out to examine whether single and extended metaphors produce differences in the levels of activation of literal-related vs. metaphor-related features associated with the metaphor vehicle. We found that during comprehension of single metaphors, metaphor-related features of the vehicle remain activated 1,000 milliseconds after metaphor comprehension while literal-related features show reduced activation, supporting the original findings of Rubio Fernández (2007). Critically, this was not the case for extended metaphors. Here, we found that literal-related features remain activated 1,000 milliseconds after the metaphor has been understood, on par with metaphor-related features. This finding supports Carston’s ‘lingering of the literal’ account because it suggests that suppression of literal-related features is reduced or may not place when understanding extended metaphors. This mechanism has been claimed to be critically engaged during the comprehension of single metaphors by Gernsbacher et al. (2001) and Rubio Fernández (2007), and the fact that suppression of literal-related features might not be at play for extended metaphors suggests a more prominent role of literal meaning in the interpretation of this type of metaphor. This may point to qualitative differences in the underlying comprehension mechanisms–e.g., in the form of different “modes of metaphor processing” as suggested by Carston (2010).

The current findings come with some caveats and are to be interpreted with caution. First of all, adapting Rubio Fernández’s paradigm to a web-based task led us to change the cross-modal priming design and present both prime and target visually. This has the limitation that we cannot be certain of the exact moment during processing in which the target word is read relative to the metaphoric prime. Though we attempted to account for this fact by setting a maximum amount of time for participants to read the metaphoric prime (4,000 milliseconds), it remains less than ideal. For a better reduction of noise, it would be necessary to run the experiment in the lab as a cross-modal priming task. A further constraint of the web-based paradigm is naturally also the reduced number of critical items used. Expanding this number would allow for better generalizability. Future research should also investigate potential differences in suppression as a function of the number of metaphoric vehicles that comprehenders are faced with. This would help answer the question of the point in time in which we would expect comprehenders to ‘switch’ from one processing mode to another (assuming that extended metaphors are actually processed differently than single ones).

Another important caveat concerns the linking assumptions used in the current experiment. In this work, we laid out an interpretation, in processing terms, of both the ‘lingering of the literal’ and ‘lingering of linguistic form’ accounts. However, these accounts are underspecified from a processing point of view, and are in theory compatible with various different empirical predictions. For example, it could be that the ‘lingering of linguistic form’ also predicts a suspension of the mechanism of suppression for literal-related features, if this view were to be interpreted differently than we have in the current work. Further work is therefore needed from both theoretical and experimental perspectives in order to thoroughly explicate the links between theory and processing and to solve the ‘puzzle’ of extended metaphors.

## Conclusion

Since the work by Carston (2010), extended metaphors have represented an interesting battle ground in the development of the relevance-theoretic view on metaphor comprehension. In the current work, we provide a new type of empirical evidence in favor of the ‘lingering of the literal’ account. Our experiment suggests that comprehenders do not suppress literal-related features when understanding extended metaphors (but they do so when understanding single metaphors). This in turn supports the idea that understanding extended metaphors involves maintaining a representation of the literal meaning of the entire metaphoric expression.

## Data availability statement

Publicly available datasets were analyzed in this study. This data can be found here: https://osf.io/eayj7/.

## Ethics statement

Ethical review and approval was not required for the study on human participants in accordance with the local legislation and institutional requirements. The patients/participants provided their written informed consent to participate in this study.

## Author contributions

CR: conceptualization, writing—original draft preparation, and writing—review and editing. IF: writing—review and editing and supervision. All authors contributed to the article and approved the submitted version.

## Funding

This paper is part of a project that has received funding from the European Research Council (ERC) under the European Union’s Horizon 2020 research and innovation program under grant agreement no. 853211 (ERC Starting Grant 2019), awarded to IF.

## Conflict of interest

The authors declare that the research was conducted in the absence of any commercial or financial relationships that could be construed as a potential conflict of interest.

## Publisher’s note

All claims expressed in this article are solely those of the authors and do not necessarily represent those of their affiliated organizations, or those of the publisher, the editors and the reviewers. Any product that may be evaluated in this article, or claim that may be made by its manufacturer, is not guaranteed or endorsed by the publisher.

## Footnotes

^1^We follow the convention within Relevance Theory to refer to *ad hoc* concepts *via* capital letters and an asterisk.

^2^ http://crr.ugent.be/programs-data/wuggy
